# Effect of antimicrobial treatments applied individually and in combination on the growth of *Listeria monocytogenes* in Queso Fresco at 3 different temperatures

**DOI:** 10.3168/jdsc.2022-0219

**Published:** 2022-07-09

**Authors:** Suneet R. Takhar, Luis A. Ibarra-Sánchez, Michael J. Miller

**Affiliations:** Department of Food Science and Human Nutrition, University of Illinois, Urbana 61801

## Abstract

Queso fresco (QF), a fresh soft cheese, is one of the most popular Hispanic cheeses in the United States and is frequently associated with *Listeria monocytogenes* outbreaks. *Listeria monocytogenes* can grow and thrive at room temperature as well as refrigeration temperatures. A combination of antimicrobial agents provides a larger spectrum of listeriostatic and listeriocidal activity resulting in a more effective approach toward the control of *L. monocytogenes*. In this study, we evaluated the efficacy of 3 Food and Drug Administration-approved generally recognized as safe (GRAS) antimicrobials, nisin (NIS), lauric arginate ethyl ester (LAE), and ε-polylysine (EPL), and the endolysin PlyP100 individually and in combination for control of *L. monocytogenes* in QF at 4°C, 7°C, and 10°C. Additionally, growth curves of *L. monocytogenes* were obtained in BHI broth and QF at these temperatures. In order for an antimicrobial to be considered a postlethality treatment for *L. monocytogenes*, it should not allow an increase of more than 2-log over the product's shelf life. Three treatments, PlyP100, PlyP100 + NIS, and EPL + LAE, effectively kept the pathogen below the 2 log growth threshold at 4°C. However, at 7°C and 10°C, none of the antimicrobial treatments could inhibit *L. monocytogenes* growth (i.e., <2 log). Overall, our results suggest the importance of considering the effect of cold storage temperatures above 4°C on the antilisterial efficacy of antimicrobial treatments in QF.

## Highlights

• A combination of antimicrobial treatments provides a more effective approach against *L. monocytogenes* growth in QF.
• PlyP100 + NIS was the most effective treatment for L. monocytogenes growth in QF.
• Listeria monocytogenes can grow up to dangerously high levels regardless of the storage temperature in untreated QF.
• EPL + LAE are good candidates to further evaluate for improving safety of QF during cold storage.
• Temperature abuse dramatically reduces the effectiveness of the tested antilisterials in QF.

## Graphical Abstract

**Figure:**
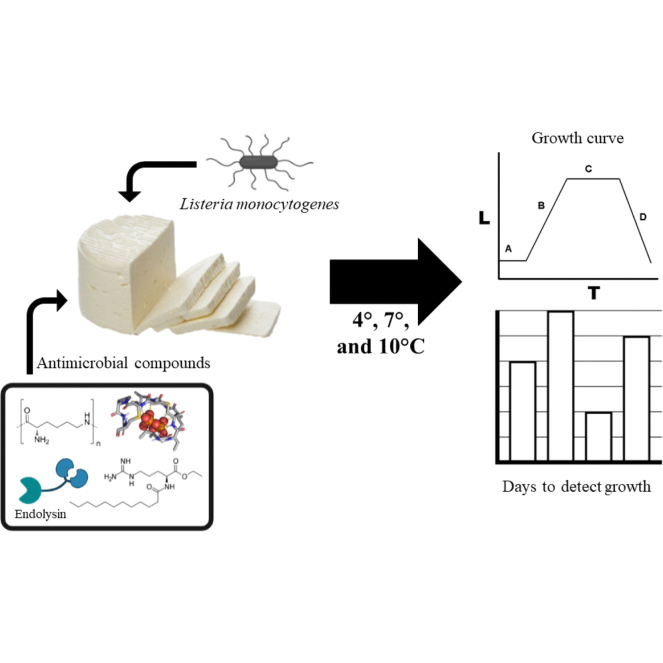
**Summary:** In this study, we evaluated the efficacy of 3 Food and Drug Administration-approved generally recognized as safe antimicrobials [nisin (NIS), lauric arginate ethyl ester (LAE), ε-polylysine (EPL), and an endolysin (PlyP100)] against growth of Listeria monocytogenes in Queso Fresco (QF) at 4°C, 7°C, and 10°C. The treatments were applied individually and in combination. Additionally, *L. monocytogenes* growth curves were achieved in brain heart infusion (BHI) broth and QF. PlyP100 + NIS was the most effective treatment and resulted in nondetectable levels of *L. monocytogenes* at prolonged storage (28 d) at 4°C. At 7°C and 10°C, none of the antimicrobial treatments could inhibit *L. monocytogenes* growth. The growth curve results indicated that L. monocytogenes can reach very high levels both in BHI broth and untreated QF regardless of storage temperature.

The ingestion of food contaminated with *Listeria monocytogenes* can cause a severe invasive illness called listeriosis that affects pregnant women, newborns, elderly, and immune-compromised individuals leading to abortion, bacteremia, sepsis, and meningoencephalitis (Farber and Losos, 1988). Due to its high mortality rate (approximately 25–30%), listeriosis is considered to be one of the most severe foodborne illnesses (Carlton et al., 2005). *Listeria monocytogenes* causes approximately 1,600 foodborne infections and 260 deaths each year in the United States (Hoffmann et al., 2015). Because *L. monocytogenes* can tolerate extreme environmental stresses, for example, pH (4.0–9.5), temperature (1 to 45°C), as well as high salt concentrations (up to 10% NaCl), it can thrive well in food processing environments (Liu et al., 2005; Friedly et al., 2008).

The Food and Drug Administration (**FDA**) has established a zero-tolerance policy for *L. monocytogenes* in ready-to-eat (**RTE**) food products, which results in huge economic losses to the dairy industry and hinders the growth of the market due to liability concerns (Van Tassell et al., 2015). Soft cheeses in particular are one of the most common food products associated with *L. monocytogenes* contamination (Guenther and Loessner, 2011; Ibarra-Sánchez et al., 2017) as factors such as high water-activity, low salt content, high pH, and storage at refrigeration temperatures favor growth of *L. monocytogenes* (Soni et al., 2010; Ibarra-Sánchez et al., 2018). Seventeen out of 58 (30%) listeriosis outbreaks reported between 1998 and 2014 were related to soft cheese and 11 out of those 17 (65%) outbreaks were linked to Latin-style cheeses (Jackson et al., 2018). Queso fresco (**QF**) is a Hispanic-style fresh, high moisture (45–55%), crumbly, salty, soft white cheese with near-neutral pH (6.0–6.5) that supports the growth of *L. monocytogenes* (Soni et al., 2012).

As *L. monocytogenes* grows and proliferates both under refrigeration and mild temperature abuse conditions, it may get transferred to foods in domestic refrigerators (Jackson et al., 2007), which would be a huge risk for RTE foods. *Listeria monocytogenes* was recovered from 1.2% out of 342 domestic refrigerators in one study (Jackson et al., 2007). In another study, *Listeria* spp. were found in 6 of 137 refrigerators (Kilonzo-Nthenge et al., 2008). A study that assessed the temperatures of 200 refrigerators in the United States demonstrated that the temperature was above the recommended 4.4°C for 33% (top shelf), 45% (middle shelf), and 80% (door) for more than 2 h per day (Godwin et al., 2007). The recommended refrigeration temperatures differ around the world but are still <7°C. A study on domestic storage malpractices in older adults pointed out the prolonged storage of RTE foods at temperatures above the recommended temperatures. The implications of these practices on growth of *L. monocytogenes* in soft cheese were tested at recommended temperature (2.5°C), slightly above recommendation (7.8°C), and ambient temperature (19.5°C; Evans and Redmond, 2019). The study revealed that longer storage at temperatures higher than recommended led to faster *L. monocytogenes* growth. In the United States, the recommended temperature is ≤4.4°C (James et al., 2017). Studies in the United States, United Kingdom, and France have indicated that food placed in open refrigerated display cases in retail undergoes temperature abuse between −1 to 16°C (Monge Brenes et al., 2020). Therefore, it becomes necessary to test the pathogen growth and antimicrobial treatments in QF at temperatures above the recommended 4.4°C.

Several FDA-approved, generally recognized as safe (**GRAS**) antimicrobials such as nisin (**NIS**), lauric arginate ethyl ester (**LAE**), and ε-polylysine (**EPL**) have exhibited antilisterial activity in milk and cheese. Nisin is a GRAS food preservative and the most commonly used bacteriocin in the food industry up to a level of 250 ppm (Gadotti et al., 2014). It is the only approved bacteriocin for use in cheese products and demonstrates broad-spectrum inhibition of gram-positive bacteria including *L. monocytogenes* (Van Tassell et al., 2015; Lourenço et al., 2017). Lauric arginate ethyl ester, a cationic surfactant, is a food preservative with a usage limit of up to 200 ppm in foods to inhibit microbial growth in cheese, meat, and poultry products (Soni et al., 2010; Ma et al., 2013, 2020). Several studies have demonstrated the effectiveness of LAE against *L. monocytogenes* in milk (Soni et al., 2010; Ma et al., 2013; Kozak et al., 2018a) and QF (Soni et al., 2010, 2012; Kozak et al., 2018b). ε-Polylysine, a homopolymer naturally produced by *Streptomyces albulus*, is a commercially available preparation that has been shown to inhibit growth of *L. monocytogenes* in milk and QF (limit 250 ppm in foods; Kozak et al., 2017, 2018a). Endolysins are the viral hydrolytic enzymes that cause the hydrolysis of the bacterial cell wall leading to cell lysis and death. Previous work in our laboratory has demonstrated the effectiveness of the endolysin PlyP100 from *L. monocytogenes* phage P100 in controlling *Listeria* in QF (Van Tassell et al., 2017; Ibarra-Sánchez et al., 2018).

A combination of antimicrobial agents provides a broader spectrum of listeriacidal and listeriostatic activity (Soni et al., 2012) and also helps reduce factors such as cost, usage limit, and likely changes in sensory properties (Kozak et al., 2017, 2018a). In this study, commercially available NIS (Danisco), LAE (CytoGaurd LA 20, A&B Ingredients Inc.), EPL (Wilshire Technologies), and PlyP100 (prepared as previously described by Van Tassell et al., 2017) were evaluated individually and in combination for their ability to control growth of *L. monocytogenes* in QF at 4°C, 7°C, and 10°C. Additionally, growth curves of *L. monocytogenes* were obtained in brain heart infusion (**BHI**) broth and QF at the given temperatures. We hypothesized that antimicrobial combinations would be effective at limiting *L. monocytogenes* growth in QF stored at the 3 selected cold temperatures.

The following strains, *Listeria innocua* ATCC 33090, *Listeria monocytogenes* NRRL B-33104, NRRL B-33513, NRRL B-33420, NRRL B-33424, and NRRL B-33419, were used in this study. The strains were grown in brain heart infusion broth (BHI; Difco) with 250 rpm agitation at 37°C for 24 h. The *L. monocytogenes* cocktail was prepared by combining equal volumes of the stationary phase cultures of 5 strains associated with foodborne outbreaks. The cocktail was serially diluted in PBS to attain 3 log_10_ cfu/mL concentration. Enumeration was conducted on PALCAM *Listeria* selective agar supplemented with 20 μg/mL ceftazidime (Tokyo Chemical Industry Co. Ltd.) and incubated at 37°C for 48 h (Ibarra-Sánchez et al., 2018). All research activities described in this publication were approved by the University of Illinois Institutional Biosafety Committee (IBC-107.1).

Miniature laboratory fresh cheese batches were prepared as described earlier (Van Tassell et al., 2015; Ibarra-Sánchez et al., 2018). Briefly, 50-mL batches of pasteurized whole milk were warmed to 35°C. The milk was then combined with rennet and CaCl_2_ and divided into 1-mL portions. Nisin (Nisaplin, Danisco) was added to the milk at this point and the tubes were returned to the water bath. The curds were inoculated with *L. monocytogenes* cocktail and set by centrifugation. The LAE, EPL, and PlyP100 were added to the drained, contaminated curds before the final pressing step. Antimicrobial treatments and their concentration (Figure 3) evaluated in QF (NIS, PlyP100, LAE, EPL, PlyP100 + NIS, and LAE + EPL) were selected based on their efficacy to inhibit *L. monocytogenes* in QF at 4°C from our previous studies (Ibarra-Sánchez et al., 2018; Martínez-Ramos et al., 2020). Cheeses were then stored at 4°C, 7°C, and 10°C for further analysis. Additionally, BHI broth was inoculated with the *L. monocytogenes* cocktail and stored at 4°C, 7°C, and 10°C to obtain growth curves.

The doubling times were calculated using a MATLAB program developed by Hoeflinger et al. (2017). The results indicated that the doubling times for *L. monocytogenes* cells were directly correlated with the storage temperature, being longest at 4°C and shortest at 10°C (Figure 1). However, the results also indicate that in case postprocessing contamination occurs, *L. monocytogenes* could grow to high numbers during prolonged storage regardless of the storage temperature. The results that we obtained for *L. monocytogenes* growth curves in QF (Figure 2) are comparable to data reported by earlier studies. A study that conducted the growth of *L. monocytogenes* in soft, semi-soft, and semi-hard artisanal cheeses reported that *L. monocytogenes* growth potential ranged between 1.8 and 4.0 log_10_ cfu/g on soft cheeses stored at 7°C for 14 d (Lahou and Uyttendaele, 2017). Similarly, our data showed that by d 14 the *L. monocytogenes* levels had increased by 4.69 log_10_ cfu/g at 7°C. Another study found that the *L. monocytogenes* population increased from initial inoculation level of 2.7 to 5.5 log_10_ cfu/g in soft cheese at the recommended storage temperature of <5°C (2.5 ± 2.2°C) at d 16 of storage and reached up to 6.8 log_10_ cfu/g at 7.8°C ± 0.4 by d 12 (Evans and Redmond, 2019). Our data indicate that the growth of *L. monocytogenes* had increased above 3 log_10_ cfu/g in 7 d at 7°C and above 4 log_10_ cfu/g by d 14 at 10°C. Soni et al. (2010) reported that the levels of *L. monocytogenes* increased by 4.3 log_10_ cfu/g (initial 4 to 8.3 log_10_ cfu/g) during 3 wk of storage of QF at 4°C. Similarly, our data indicated a growth of 4.71 log_10_ cfu/g at d 21 of storage at 4°C.

**Figure 1:**
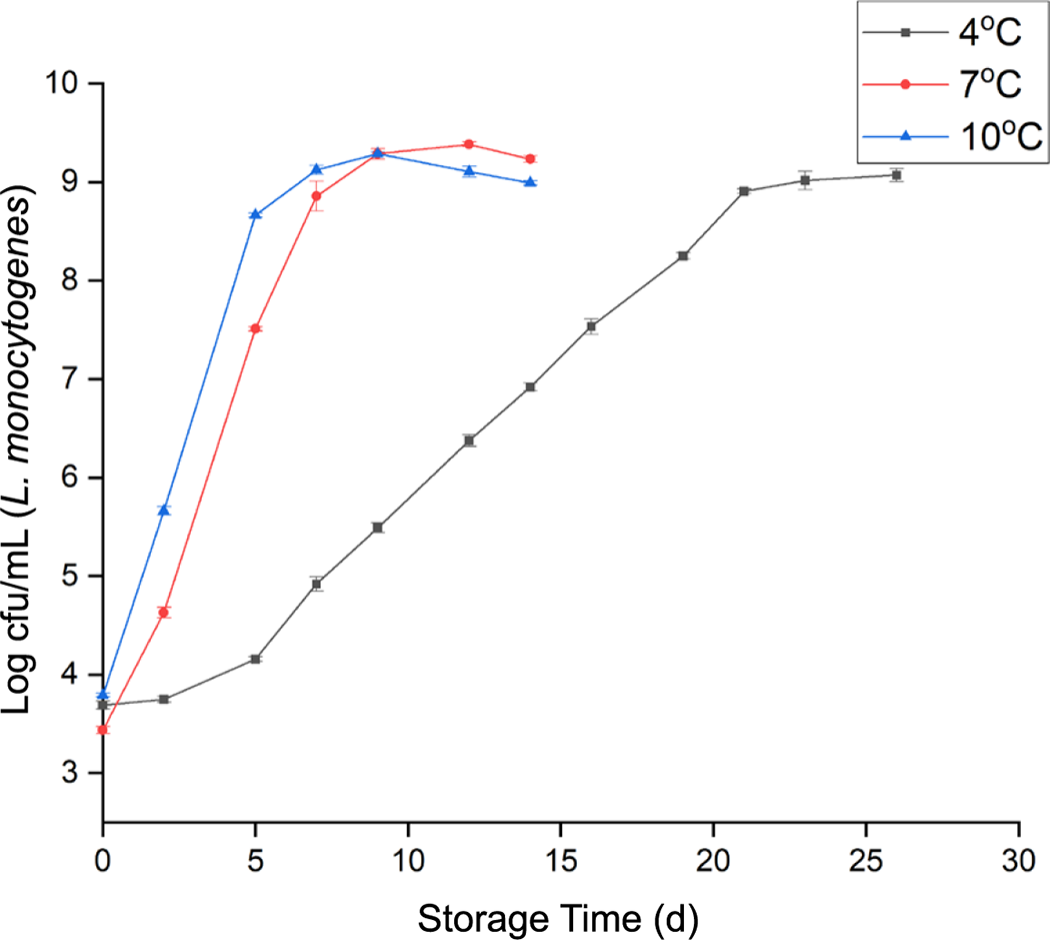
*Listeria monocytogenes* growth curves in brain heart infusion broth. Doubling time was 43.37 ± 1.25, 11.34 ± 0.32, and 9.84 ± 0.14 h at 4°C, 7°C, and 10°C, respectively. Values are means ± SEM of 3 independent experiments.

**Figure 2:**
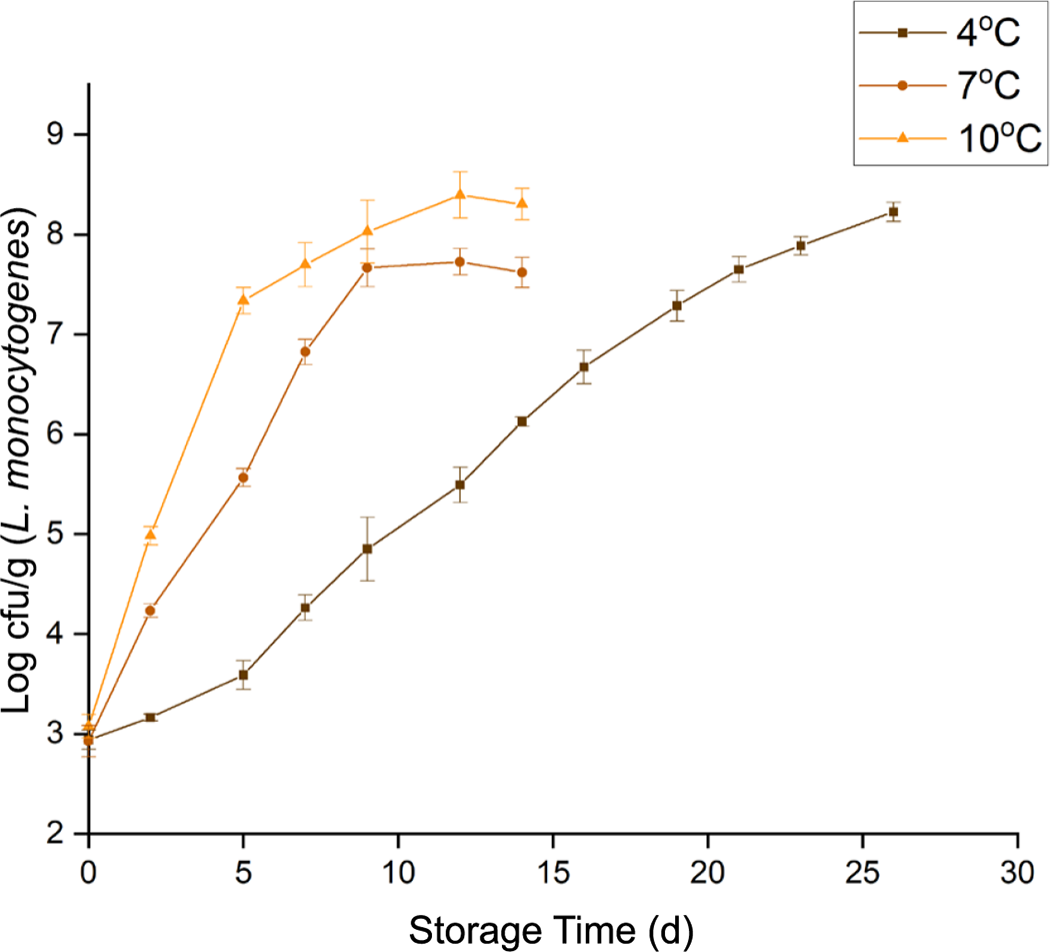
*Listeria monocytogenes* growth curves in Queso Fresco cheese. Doubling time was 47.53 ± 1.14, 20.32 ± 1.4, and 12.84 ± 0.94 at 4°C, 7°C, and 10°C, respectively. Values are means ± SEM of 3 independent experiments.

An antimicrobial application could be considered a postlethality if it could reduce at least 1 log cfu *L. monocytogenes* in a product before it leaves the facility and would not allow more than a 2 log increase in *L. monocytogenes* levels over the product's shelf life (USDA-FSIS, 2014; Kozak et al., 2018b). Four antimicrobials, NIS, LAE, EPL, and PlyP100, were tested for their efficacy in keeping *L. monocytogenes* levels in QF below the 2 log threshold. Our results indicated that only 3 treatments, PlyP100, PlyP100 + NIS, and EPL + LAE, were effective to control the pathogen below the 2 log growth threshold at 4°C (Figure 3). The efficacy of PlyP100 by itself and in combination with NIS against the growth of *L. monocytogenes* in QF has been previously demonstrated in our laboratory (Van Tassell et al., 2017; Ibarra-Sánchez et al., 2018). The combination exhibited strong synergism and resulted in nondetectable levels of *L. monocytogenes* after storage of QF at 4°C for 28 d. The synergy between PlyP100 and NIS may be the result of both antimicrobials targeting the cell wall such that NIS pore formation and PlyP100 peptidoglycan hydrolysis resulted in enhanced lysis of *L. monocytogenes* cells. Alternatively, the listeriostatic effect of PlyP100 may complement the gradual loss of NIS in QF in that residual NIS in QF reduce *L. monocytogenes* populations, whereas PlyP100 prevents regrowth of survivors (Ibarra-Sánchez et al., 2018). A previous study has also reported that the combination of EPL + LAE works well as bacteriostatic against *L. monocytogenes* in QF at 4°C (Martínez-Ramos et al., 2020). Although the aforementioned treatments (PlyP100, PlyP100 + NIS, and EPL + LAE) can limit *L. monocytogenes* growth in QF to less than 2 log, only PlyP100 + NIS may comply with the FDA zero-tolerance policy of *L. monocytogenes* due to its efficacy in reducing *L. monocytogenes* populations in QF. Also, whereas *L. monocytogenes* infectious dose may be estimated as low as 10° to 10^4^ cfu/g (Busch et al., 2022) and contamination levels of less than 100 cfu/g in fresh cheeses have been observed (USDA-FSIS, 2003), antimicrobial treatments that limit *L. monocytogenes* growth over QF shelf life (e.g., PlyP100 and EPL + LAE) can contribute to reducing the risk of *L. monocytogenes* infection in situations where QF is contaminated with the pathogen.

**Figure 3:**
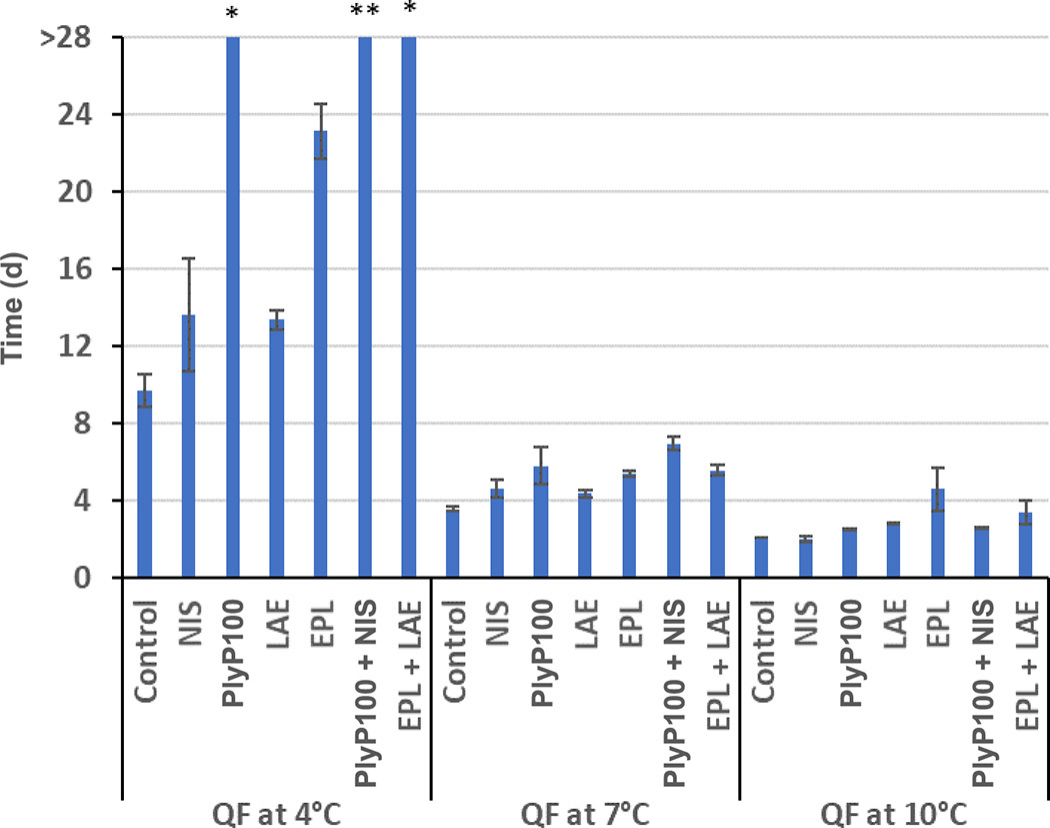
Required time for 2 log growth of *Listeria monocytogenes* in Queso Fresco (QF) stored at 4°C, 7°C, and 10°C. NIS = nisin (250 µg/g); PlyP100 = PlyP100 (2.5 U/g); LAE = lauric arginate (200 µg/g); EPL = ε-polylysine (250 µg/g); PlyP100 + NIS = 2.5 U/g PlyP100 + 250 µg/g nisin; EPL + LAE = 250 µg/g ε-polylysine + 66.66 µg/g lauric arginate. *Listeria monocytogenes* inhibition over 28 d of QF shelf life: <2 log growth (*); >1 log reduction (**). One U = amount of enzyme necessary to decrease the optical density at 600 nm (OD_600_) of *Listeria* cells in suspension by 0.01/min. Values are means ± SEM.

As reported by earlier studies, there is a possibility of temperature abuse during shipping, handling, and storage. Consequently, the efficacy of the antimicrobials was also tested at 7°C and 10°C. At 7°C and 10°C, none of the antimicrobial treatments could inhibit *L. monocytogenes* growth (<2 log). However, some treatments were able to delay the growth at these temperatures. At 7°C, 3 extra days relative to control were required for cheeses containing PlyP100 and PlyP100 + NIS to achieve 2 log growth. At 10°C, 3 extra days relative to control were required for cheeses supplemented with EPL.

Earlier research has demonstrated that treatment of QF with LAE leads to an initial reduction of *L. monocytogenes*, but regrowth occurs during storage at 4°C for longer periods of time (Soni et al., 2010). Kozak et al. (2018a) reported that following the initial reduction of *L. monocytogenes* in QF, the counts returned to initial inoculation levels by d 7 at 7°C. Kozak et al. (2018a) also reported that EPL alone did not make a significant difference on the growth of *L. monocytogenes* and that the treatment with combination of LAE + EPL did not lead to a significant difference from treatment with LAE by itself at 7°C. Nisin alone when used at the permissible levels of 250 ppm does not provide sufficient barrier against growth of *L. monocytogenes* in QF, which may be due to the lack of stability of NIS at near-neutral pH (Ibarra-Sánchez et al., 2020).

The growth curves in our study indicate that without any treatment *L. monocytogenes* could grow in QF up to high levels that are unsafe for human consumption regardless of the cold storage temperature. The results also support our previous findings that PlyP100 + NIS effectively control *L. monocytogenes* during storage at 4°C. Additionally, EPL + LAE seem to be good candidates for further research in improving the safety of QF during cold storage. Future work is needed to explore antilisterial interventions that effectively inhibits *L. monocytogenes* in QF under cold temperature abuse conditions.
